# Clinical and Healthcare Disparities in Migrant Patients with Parkinson’s Disease: A Matched Cohort Study in a Multiethnic European Population

**DOI:** 10.3390/jcm15145528

**Published:** 2026-07-15

**Authors:** Alejandro Peral-Quirós, Núria Caballol, Anna Planas-Ballvé, Paula Lombardo-del Toro, Isabel Gómez-Ruiz, Marta Balagué-Marmaña, Cristian Pérez-Pellejero, Cristina Fornés, Asunción Ávila-Rivera

**Affiliations:** 1 Movement Disorders Unit, Neurology Department, Complex Hospitalari Universitari Moisès Broggi, Carrer d’Oriol Martorell, 12, 08970 Sant Joan Despí, Spain; aperalq@csi.cat (A.P.-Q.); ncaballol@csi.cat (N.C.); annaplanasb@gmail.com (A.P.-B.); plombardodt@csi.cat (P.L.-d.T.); migomez@csi.cat (I.G.-R.); mbalague@csi.cat (M.B.-M.); cperezpe@csi.cat (C.P.-P.); cfornesg@csi.cat (C.F.); 2 Department of Medicine, Faculty of Medicine and Health Sciences, University of Barcelona, 08036 Barcelona, Spain

**Keywords:** Parkinson’s disease, immigrant populations, ethnicity, motor phenotype, treatment adherence, healthcare disparities

## Abstract

**Background/Objectives**: Data on Parkinson’s disease in immigrant populations are limited despite increasing migration and population aging in Europe. This study aimed to evaluate differences in clinical presentation and healthcare engagement between immigrant and native patients with Parkinson’s disease in a multiethnic European setting. **Methods**: We conducted a retrospective matched cohort study including immigrant patients with Parkinson’s disease followed at our Movement Disorders Unit between 2000 and 2025. Immigrant patients were matched 1:1 with native patients by age, sex, and year of diagnosis. Clinical characteristics, prodromal symptoms, motor severity, and healthcare engagement variables were analyzed. **Results**: Ninety-eight immigrant patients were matched with 98 native patients. Most immigrants originated from Latin America and North Africa. Native patients showed a higher prevalence of hyposmia than immigrant patients (49.0% vs. 26.5%; *p* = 0.002). No significant differences were observed in disease duration, pattern of motor symptom onset, motor severity, Hoehn and Yahr stage, MDS-UPDRS scores, or antiparkinsonian treatment during follow-up, although native patients had higher postural tremor scores at baseline. Immigrant patients demonstrated lower healthcare engagement, with higher rates of missed follow-up visits (34.7% vs. 16.3%; *p* = 0.003) and poorer treatment adherence (24.5% vs. 11.2%; *p* = 0.016). **Conclusions**: Immigrant patients with Parkinson’s disease showed a lower prevalence of hyposmia and reduced healthcare engagement despite otherwise comparable clinical characteristics, motor severity, and treatment patterns. These findings highlight the importance of considering sociocultural determinants and barriers to healthcare utilization when managing Parkinson’s disease in increasingly diverse populations.

## 1. Introduction

Population aging is associated with an increased prevalence of neurodegenerative disorders, including Parkinson’s disease (PD). Over recent decades, Europe has experienced a marked rise in migratory movements, and although most individuals migrate at younger ages, immigrant populations progressively settle and age within host countries. Few European studies have evaluated the incidence of PD in immigrant populations; however, available evidence suggests that it may represent a relevant challenge for European healthcare systems [1,2].

The healthcare area covered by our institution, primarily encompassing the cities of L’Hospitalet de Llobregat and Cornellà de Llobregat, has undergone a substantial increase in population diversity over the last two decades, becoming one of the most multiethnic urban settings in Catalonia (Spain). The population of L’Hospitalet de Llobregat increased from 241,782 inhabitants in 2000 to 292,161 in 2025. During this period, the proportion of immigrant residents rose from 2.44% to 27.34%, of whom 6.36% were older than 59 years. Most immigrants originated from the Americas (54.79%, mainly South America), followed by Asia (21.78%, predominantly South Asia), Europe (12.01%, mostly from the European Union), and Africa (11.40%, mainly North Africa). In Cornellà de Llobregat, the population increased from 80,998 inhabitants in 2000 to 92,255 in 2025, while the immigrant population rose from 1.99% to 18.48%. Of these, 7.58% were older than 59 years. Approximately 49.53% originated from the Americas (mainly South America), 19.04% from Africa (primarily North Africa), 18.59% from Europe (mostly from the European Union), and 12.82% from Asia (predominantly Southeast Asia) [3].

In general, immigrant populations may exhibit health profiles that differ from those of native populations. These differences are influenced by the migration process itself, as well as by social and structural determinants in the host country, including migration policies, access to healthcare systems, and cultural norms [4,5].

There is very limited evidence regarding the clinical characteristics of PD in immigrant populations, and most studies have focused primarily on cognitive assessment [6,7,8]. The incidence and clinical presentation of neurodegenerative diseases in immigrant populations may differ from those observed in the majority population of the host country. Factors such as geographic origin, genetic background, sex, and age may contribute to these differences. Furthermore, language, cultural, and educational barriers may hinder symptom recognition, access to healthcare services, and clinical or cognitive assessment, potentially affecting diagnosis, treatment, and long-term follow-up. Moreover, although healthcare coverage in our setting is universally available to both immigrants and native residents, equal access does not necessarily translate into equity in health outcomes, as barriers related to communication, cultural differences, or logistical limitations may still persist.

In this context, and given the substantial increase in the number of immigrant patients with PD, we aimed to evaluate whether migratory status is associated with differences in clinical presentation and healthcare participation among patients with PD in a metropolitan area of the Spanish healthcare system characterized by a high immigration rate.

## 2. Materials and Methods

### 2.1. Study Population

The PD database of the Movement Disorders Unit at Complex Hospitalari Universitari Moisès Broggi (Sant Joan Despí, Barcelona, Spain) was retrospectively reviewed to identify all consecutive patients diagnosed with PD between January 2000 and December 2025. A total of 1213 patients were identified, including 98 immigrant patients (8.1%).

Because immigrant patients were significantly younger at diagnosis than native patients (62.99 ± 11.23 vs. 72.50 ± 8.39 years, *p* < 0.00001), direct comparisons between the two groups would have been substantially confounded by age.

**Figure 1:**
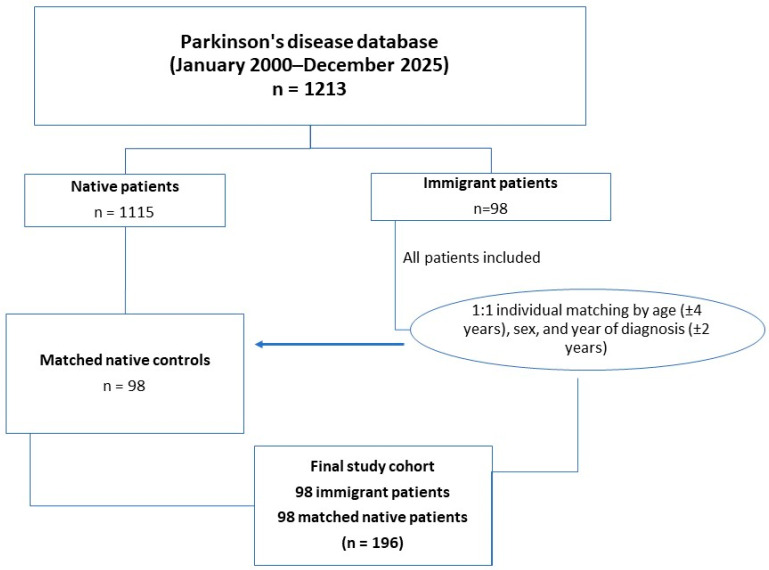
Flow diagram of patient selection for the retrospective matched cohort study.

Therefore, this retrospective matched cohort study included all immigrant patients and a 1:1 sample of native patients individually matched by age (±4 years), sex, and year of diagnosis (±2 years), yielding 98 matched pairs (Figure 1). Matching was performed before statistical analysis using individual pairwise matching without replacement; once a native patient had been selected as a control, that individual was not eligible for matching with another immigrant patient. Age and sex were selected because they are major determinants of clinical phenotype and disease progression. Year of diagnosis was included to account for temporal changes in clinical practice and the availability of antiparkinsonian therapies during the 25-year study period.

### 2.2. Clinical Assessment

Baseline (V1) clinical characteristics and data from the last available follow-up visit (V2) were compared between the matched groups. Variables included prodromal symptoms, family history, exposure to dopamine-blocking agents, initial motor presentation, Hoehn and Yahr stage, MDS-UPDRS parts I–IV, and levodopa equivalent daily dose (LEDD).

Healthcare engagement was evaluated using two indicators: (1) failure to attend at least one scheduled follow-up visit and (2) medication adherence errors during follow-up. Missed follow-up was defined as failure to attend at least one scheduled outpatient visit to the Movement Disorders Unit. Medication adherence errors during follow-up were defined as any documented deviation from the prescribed antiparkinsonian treatment regimen, including missed doses, incorrect dosing, and treatment discontinuation when not attributable to adverse effects. Thus, treatment discontinuation was considered one type of medication adherence error rather than a separate outcome. Because follow-up duration varied among patients, disease duration at the last available visit was included as a covariate in the multivariable analyses.

### 2.3. Sample Size

No formal sample size calculation was performed because this was a retrospective observational study including all immigrant patients available in the institutional database during the study period. Consequently, the study should be considered exploratory and hypothesis-generating.

### 2.4. Statistical Analysis

Continuous variables were assessed for normality using the Shapiro–Wilk test and are presented as mean ± standard deviation (SD) or median (interquartile range), as appropriate. Categorical variables are expressed as frequencies and percentages. Because of the matched design, continuous variables were compared using the paired Student’s *t*-test or Wilcoxon signed-rank test, as appropriate, whereas paired categorical variables were analyzed using McNemar’s test. Multivariable binary logistic regression models were used to evaluate whether immigrant status was independently associated with loss to follow-up and medication adherence errors after adjustment for age at diagnosis, sex, and disease duration. Results are presented as adjusted odds ratios (ORs) with 95% confidence intervals (CIs). No adjustment for multiple comparisons was performed because secondary analyses were considered exploratory. All tests were two-sided, with *p* < 0.05 considered statistically significant. Statistical analyses were performed using IBM SPSS Statistics version 26 (IBM Corp., Armonk, NY, USA).

### 2.5. Ethical Approval and Informed Consent

The study was approved by the local ethics committee (Bellvitge Hospital, No PR 163/24) and written informed consent was obtained from all participants before their data was included in the study’s Parkinson’s disease database. The study was conducted in accordance with the ethical principles of the Declaration of Helsinki.

## 3. Results

### 3.1. Demographic Characteristics and Ethnic Distribution of the Cohort

We reviewed 98 immigrant patients with PD, matched by age (±4 years), sex, and year of diagnosis (±2 years), with 98 native patients with PD (Table 1). Most immigrant patients originated from Latin America (62.24%, *n* = 61), including individuals from Peru (*n* = 28), Ecuador (*n* = 15), Venezuela (*n* = 7), Bolivia (*n* = 5), Colombia (*n* = 2), Paraguay (*n* = 2), Honduras (*n* = 1), and Uruguay (*n* = 1). Patients from North Africa constituted the second largest subgroup (20.41%, *n* = 20), including 19 individuals from Morocco and one from Algeria. Other ethnic minority groups included patients from the Caribbean (7.14%), Europe (5.10%), and Asia (3.06%).

Given that Latin American patients represented the majority of the immigrant cohort, whereas the remaining ethnic groups were small and heterogeneous, comparisons between native patients and ethnic subgroups, as well as comparisons among ethnic subgroups, were not performed because of insufficient statistical power and limited interpretability.

### 3.2. Temporal Trends in Parkinson’s Disease Diagnosis Among Immigrant Patients

Analysis of the year of PD diagnosis among immigrant patients revealed only isolated cases during the initial study period (2000–2010), including a single case diagnosed in 2000 and sporadic cases recorded between 2007 and 2010. Between 2011 and 2021, the number of new diagnoses progressively increased, ranging from two to six cases per year. The most pronounced rise was observed from 2022 onward, reaching up to 19 new diagnoses annually. This increase likely reflects the parallel growth of the immigrant population and the cumulative aging of migrant communities, although improved access to healthcare services among immigrant populations may also have contributed.

To further assess temporal trends, a simple linear regression analysis was performed using calendar year as the independent variable and the annual number of PD diagnoses as the dependent variable. The analysis showed a positive slope (β = 1.57), indicating an average yearly increase in the number of diagnoses. The correlation coefficient was moderate (r = 0.56), and the coefficient of determination (R^2^ = 0.31) suggested that approximately 31% of the observed variability could be explained by temporal progression. However, the association did not reach statistical significance (*p* = 0.25).

### 3.3. Clinical Characteristics at Baseline (V1) and Follow-Up (V2)

Baseline and follow-up clinical characteristics of immigrant and native patients are summarized in Table 1 and Table 2, respectively.

No significant differences were observed between groups regarding family history of PD. Prior exposure to dopamine-blocking drugs was more frequent among native patients, reaching marginal statistical significance (11.22% vs. 4.08%, *p* = 0.05).

Regarding premotor symptoms at the first visit, native patients exhibited a significantly higher prevalence of hyposmia than immigrant patients (48.98% vs. 26.53%, *p* = 0.002), whereas no significant differences were observed for other premotor manifestations, including REM sleep behavior disorder, constipation, depression, or minor hallucinations.

No significant differences were observed in the pattern of motor symptom onset at V1. Disease duration did not differ significantly between immigrant and native patients either at baseline or at follow-up. Similarly, no significant differences were observed in motor severity as assessed by the MDS-UPDRS and Hoehn and Yahr scales (Table 1 and Table 2).

Motor performance was further evaluated using specific tremor, bradykinesia, rigidity, and combined rigidity-plus-bradykinesia (R+B) scores derived from the MDS-UPDRS Part III. No significant differences were observed between the groups regarding bradykinesia, rigidity, or combined R+B scores. In contrast, native patients exhibited a significantly higher postural tremor score at V1 than the matched immigrant cohort (t (97) = 3.05, *p* = 0.003), 95% CI [0.17, 0.81]).

No significant differences were observed in antiparkinsonian treatment, including medication type or levodopa-equivalent dosage, throughout the follow-up period (Table 1 and Table 2).

### 3.4. Treatment Adherence and Follow-Up Attendance

Analysis of longitudinal follow-up revealed significant differences in healthcare attendance and treatment adherence between groups. Immigrant patients missed significantly more scheduled follow-up visits and demonstrated a higher frequency of medication adherence errors than native patients. However, no differences were observed in treatment intolerance to the main antiparkinsonian therapies, including monoamine oxidase inhibitors, levodopa, dopamine agonists, and catechol-O-methyltransferase inhibitors (Table 2).

Binary logistic regression analyses were performed to evaluate whether immigrant status was independently associated with loss to follow-up and medication adherence errors after adjustment for age at diagnosis, sex, and disease duration. Immigrant status remained independently associated with both loss to follow-up (adjusted OR 2.69, 95% CI 1.29–5.59; *p* = 0.008) and medication adherence errors (adjusted OR 2.69, 95% CI 1.20–6.06; *p* = 0.017). Age at diagnosis, sex, and disease duration were not independently associated with either outcome. Both models showed adequate goodness of fit according to the Hosmer–Lemeshow test (*p* = 0.97 and *p* = 0.47, respectively).

## 4. Discussion

There are few studies addressing PD in immigrant populations [1,2,6,7,8,9,10,11,12,13]. The first attempt to explore the public health relevance of PD among migrants in Europe [1] demonstrated that, similar to our setting, approximately 8% of all PD cases in Europe (129,645 out of an estimated total of 1,567,835 cases) occur in immigrant populations, representing a potentially relevant challenge for European healthcare systems. Furthermore, this issue is expected to gain increasing importance in the future due to sociodemographic changes driven by rising immigration rates and the aging of migrant populations [1].

In our cohort, the annual number of PD diagnoses among immigrants showed an upward pattern over the study period; however, this trend did not reach statistical significance, likely reflecting the limited sample size and year-to-year variability. Therefore, our findings should be interpreted cautiously and cannot be considered evidence of a significant temporal increase. Nevertheless, in the context of the well-established demographic evolution of European migrant populations [1,3], the absolute number of immigrants living with PD and requiring neurological care is expected to increase in the coming decades, making this an increasingly relevant issue for routine clinical practice.

Another notable finding was the higher frequency of prior exposure to dopamine-blocking drugs among native patients, although this difference reached only marginal statistical significance. This observation may reflect differences in healthcare utilization, prescribing patterns, or the prevalence and recognition of psychiatric or gastrointestinal conditions for which these medications are commonly prescribed, rather than differences directly related to PD itself. However, given the limited sample size and the exploratory nature of this analysis, this finding should be interpreted cautiously and warrants further investigation in larger studies.

Relatively few studies have evaluated non-motor symptoms across ethnically diverse populations with PD, and, to our knowledge, none have specifically examined prodromal symptoms. Previous research suggests that the prevalence of several non-motor manifestations varies across ethnic groups, including excessive daytime sleepiness, gastrointestinal symptoms, restless legs syndrome, depression, impulse control disorders, and cognitive impairment, likely reflecting a combination of genetic, environmental, cultural, and treatment-related factors [14,15,16,17,18,19].

In our study, hyposmia—a characteristic prodromal feature of PD—was less frequently reported among immigrant patients than native patients. This finding may reflect cultural differences in symptom perception and reporting, as well as environmental or dietary influences, although information bias related to clinical history taking cannot be excluded. Given the limited sample size, these results should be interpreted with caution, and future studies using standardized tools for prodromal symptom assessment are warranted.

Previous studies have also suggested that the motor phenotype of PD may differ across ethnic populations, with variations in tremor, bradykinesia, rigidity, and response to levodopa, likely reflecting a combination of genetic and environmental factors [14,17,19,20]. In the present study, native patients exhibited higher postural tremor scores at baseline than matched immigrant patients. Although evidence specifically addressing ethnic differences in postural tremor is limited, this finding may reflect differences in disease phenotype, genetic or environmental influences, or variability in symptom recognition and clinical assessment. However, given the exploratory nature of this analysis and the modest sample size, this observation should be interpreted cautiously and confirmed in larger, ethnically diverse cohorts.

Comparisons between native patients and ethnic subgroups, as well as among ethnic subgroups, were not performed because the immigrant cohort was predominantly composed of Latin American patients (62.24%), whereas the remaining ethnic groups were represented by small and heterogeneous samples, precluding meaningful statistical analyses. Larger studies with more balanced ethnic representation are needed to clarify the influence of ethnicity on the clinical phenotype and progression of PD.

Importantly, analysis of follow-up attendance and treatment adherence throughout longitudinal follow-up demonstrated significant differences between groups. Immigrant patients showed a higher frequency of missed scheduled visits and poorer pharmacological adherence than native patients. After adjustment for age at diagnosis, sex, and disease duration, immigrant status remained independently associated with both reduced healthcare follow-up and poorer treatment adherence. These differences do not appear to be related to poorer medication tolerance, as no differences were observed in intolerance to the main therapeutic agents used, including monoamine oxidase-B (MAO-B) inhibitors, levodopa, dopamine agonists, and catechol-O-methyltransferase (COMT) inhibitors. The mechanisms underlying these findings were not evaluated in the present study. However, they may reflect factors that have been described in previous studies of healthcare disparities, such as socioeconomic disadvantage, language barriers, cultural differences in health beliefs, difficulties accessing healthcare services, occupational constraints, or stigma associated with chronic neurological diseases. These potential explanations should be considered hypothesis-generating rather than causal interpretations and warrant further investigation in prospective studies specifically designed to assess these factors. Overall, our findings highlight the need for future research to identify the determinants of reduced healthcare follow-up and treatment adherence among immigrant patients with PD, which may ultimately inform targeted interventions to improve continuity of care.

Another limitation is that multiple statistical comparisons were performed without formal correction for multiple testing. Consequently, some statistically significant findings may represent false-positive results and should be interpreted cautiously. Nevertheless, the main findings regarding loss to follow-up and treatment discontinuation were further evaluated using multivariable logistic regression analyses, which supported the robustness of these associations.

## 5. Conclusions

This study highlights the growing clinical relevance of PD among immigrant populations in metropolitan areas with high migratory influx. In our cohort, immigrant patients represented an increasing proportion of patients followed over time, reflecting the evolving demographic profile of European healthcare systems.

Overall, immigrant and native patients showed comparable disease duration, motor symptom onset, motor severity, and antiparkinsonian treatment throughout follow-up. Native patients had a higher prevalence of hyposmia at presentation, a finding that remained consistent in the Latin American subgroup, whereas other premotor manifestations did not differ significantly between groups. Although native patients showed higher postural tremor scores at baseline, no consistent differences in overall motor severity or progression were identified.

Despite similar clinical characteristics, immigrant patients demonstrated lower healthcare engagement, with more missed follow-up visits and poorer treatment adherence. These findings suggest that universal healthcare coverage alone may not ensure equitable healthcare utilization. Sociocultural, linguistic, and socioeconomic barriers may contribute to these disparities, although these factors were not directly assessed in this study.

Overall, our findings support the need to consider migratory and sociocultural factors when planning the long-term care of patients with PD. Future multicenter studies including larger and more diverse immigrant populations are needed to confirm these observations and to better understand the determinants of healthcare engagement and clinical presentation in this population.

## Figures and Tables

**Table 1:** Baseline demographic and clinical characteristics of immigrant and native patients with Parkinson’s disease (V1).

Variable	Immigrant Patients (*n* = 98)	Native Patients (*n* = 98)	*p* Value *
**Demographic characteristics**			
Age, years mean ± SD (range)	62.99 ± 11.23 (36–87)	63.05 ± 10.67 (40–88)	Matched
Male sex, *n* (%)	55 (56.1)	55 (56.1)	Matched
**Clinical characteristics**			
Disease duration (months) mean ± SD	37.51 ± 45.42	33.65 ± 40.06	0.54
Family history positive *n* (%)	11 (11.34)	17 (17.35)	0.23
Prior exposure to dopamine-blocking drugs, *n* (%)	4 (4.08)	11 (11.22)	**0.05**
**Premotor symptoms *n* (%)**			
Hyposmia	26 (26.53)	48 (48.98)	**0.002**
REM sleep behavior disorder	29 (29.59)	31 (31.63)	0.73
Constipation	47 (47.96)	37 (37.76)	0.15
Depression	29 (29.59)	27 (27.55)	0.75
Minor hallucinations	13 (13.27)	8 (8.16)	0.32
**Disease severity**			
Hoehn and Yahr stage median (range)	2 (1–4)	2 (1–4)	0.92
MDS-UPDRS I mean ± SD	5.28± 5.08	6.11 ± 6.67	0.31
MDS-UPDRS II mean ± SD	8.48 ± 6.10	6.79 ± 6.52	0.08
MDS-UPDRS III mean ± SD	20.38 ± 13.89	20.88 ± 13.71	0.79
MDS-UPDRS IV mean ± SD	0.31 ± 1.37	0.32 ± 1.44	0.96
LEDD V1 mean ± SD	317.11 ± 312.78	266.18 ± 311.65	0.25

MDS-UPDRS = Movement Disorders Society-Unified Parkinson Disease Rating Scale. LEDD = Levodopa Equivalent Daily Dose. * Statistical significance was assessed using the paired Student’s *t*-test for normally distributed continuous variables, the Wilcoxon signed-rank test for non-normally distributed variables, and McNemar’s test for paired binary variables. Statistically significant *p* values (*p* < 0.05) are shown in **bold**.

**Table 2:** Longitudinal clinical assessment, treatment adherence, and follow-up attendance in immigrant and native patients with Parkinson’s disease at the last follow-up visit (V2).

Variable	Immigrant Patients (*n* = 98)	Native Patients (*n* = 98)	*p* Value *
**Demographic characteristics**			
Age. years mean ± SD (range)	66.27 ± 11.08 (36–89)	66.90 ± 10.51 (43–88)	0.11
Male sex, *n* (%)	55 (56.1)	55 (56.1)	Matched
**Clinical characteristics**			
Disease duration (months) mean ± SD	74.72 ± 58.03	82.24 ± 60.12	0.90
**Disease severity**			
Hoehn and Yahr stage median (range)	2 (0–5)	2 (0–4)	0.25
MDS-UPDRS III mean ± SD	16.63 ± 14.859	19.14 ± 13.507	0.24
MDS-UPDRS IV mean ± SD	0.83 ± 2.50	0.41 ± 1.23	0.19
LEDD mean ± SD	591.76 ± 597.99	709.96 ± 622.08	0.09
**Follow-up and treatment adherence**			
Patients missing ≥ 1 follow-up visit, *n (%)*	34 (34.69)	16 (16.33)	**0.008**
Patients with medication-taking errors, *n (%)*	24 (24.49)	11 (11.22)	**0.017**
Patients with treatment intolerance *n (%)*	22 (22.45)	25 (25.51)	0.62

MDS-UPDRS = Movement Disorders Society-Unified Parkinson Disease Rating Scale. LEDD = Levodopa Equivalent Daily Dose. * Statistical significance was assessed using the paired Student’s *t*-test for normally distributed continuous variables, the Wilcoxon signed-rank test for non-normally distributed variables, and McNemar’s test for paired binary variables. Statistically significant *p* values (*p* < 0.05) are shown in **bold**.

## Data Availability

The data are unavailable due to privacy or ethical restrictions.
